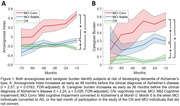# Anosognosia and caregiver distress independently identify subjects at high risk of developing dementia of Alzheimer’s type

**DOI:** 10.1002/alz.086637

**Published:** 2025-01-09

**Authors:** Dimitrios Pantazis, Takfarinas Medani, Anand A Joshi, Swapna Mahurkar‐Joshi, Richard Leahy, Katia Andrade

**Affiliations:** ^1^ Massachusetts Institute of Technology, Cambridge, MA USA; ^2^ University of California, Los Angeles, CA USA; ^3^ University of Southern California, Los Angeles, CA USA; ^4^ University of California Los Angeles, Los Angeles, CA USA; ^5^ Brain‐Computer Interfaces team, ESPCI Paris / PSL Research University, Paris France; ^6^ FrontLab, Paris Brain Institute, Salpêtrière University Hospital, Paris France; ^7^ Institute of Memory and Alzheimer’s Disease (IM2A), Salpêtrière University Hospital, Paris France

## Abstract

**Background:**

It is estimated that up to 80% of people with Alzheimer’s disease (AD) may have some form of anosognosia. Anosognosia also constitutes a major source of stress for caregivers as it delays diagnosis and affects compliance with treatment. Here, we aimed to explore whether and how early anosognosia and caregiver burden could independently serve as indicators for identifying patients at risk of converting to AD.

**Method:**

We analyzed data from 1473 participants enrolled in the ADNI longitudinal study. Participants were stratified into three distinct groups: the Mild Cognitive Impairment Conversion group (MCI Conv) comprising n=175 patients who progressed to AD during the study, the Mild Cognitive Impairment Stable group (MCI Stable) comprising n=531 patients who maintained stability throughout the study, and the Cognitive Normal group (CN) comprising n=767 individuals. For each individual and study visit, anosognosia assessment was based on the overall participant versus partner discrepancy score of the Everyday Cognition (ECog) scale. Caregiver burden was based on the Neuropsychiatric Inventory (NPI).

**Result:**

We found that both anosognosia index and caregiver burden could robustly identify patients at risk of converting to AD (Figure 1). Anosognosia index was significantly more positive for the MCI Conv than the MCI Stable group as early as 48 months before conversion (t = 2.58, p = 0.0163, FDR‐adjusted). Caregiver burden was significantly heightened in the MCI Conv than the MCI Stable group as early as 36 months before conversion (t = 2.24, p = 0.026, FDR‐adjusted). We obtained similar caregiver burden robust effects differentiating the MCI Stable and CN groups for at least 36 months of stable condition (t=2.40, p=0.0256, FDR‐adjusted), though anosognosia index was able to differentiate the two groups for a shorter 12‐month period (t=2.80, p=0.005, FDR‐adjusted).

**Conclusion:**

Anosognosia emerged as a differentiator as early as 4 years before conversion, while caregiver burden proved effective in distinguishing individuals at risk 3 years prior to the onset of conversion. These results underscore the significance of these measures in early detection and provide valuable insights for proactive interventions in the trajectory of Alzheimer's disease.